# The effects of melatonin on the concentrations of inflammatory cytokines and proteins, serotonin, cortisol and melatonin in ovariohysterectomised female dogs

**DOI:** 10.1002/vms3.1112

**Published:** 2023-03-13

**Authors:** Sina Salavati, Asghar Mogheiseh, Saeed Nazifi, Atefeh Amiri, Behrooz Nikahval

**Affiliations:** ^1^ Department of Clinical Sciences School of Veterinary Medicine Shiraz University Shiraz Fars Iran

**Keywords:** anaesthesia, inflammation, melatonin, reproductive tract, stress

## Abstract

**Background:**

Ovariohysterectomy (OHE) induces inflammation and stress in female dogs. The anti‐inflammatory effects of melatonin have been reported in several studies.

**Objectives:**

The goal of this study was to assess the effects of melatonin on the concentrations of melatonin, cortisol, serotonin, α‐1‐acid glycoprotein (AGP), serum amyloid A (SAA), c‐reactive protein (CRP), interleukin‐10 (IL‐10), interleukin‐8 (IL‐8), interleukin‐1β (IL‐1β) and tumour necrosis factor‐α (TNF‐α) before and after OHE.

**Methods:**

The total number of animals was 25 and aligned in 5 groups. Fifteen dogs were divided into three groups (*n* = 5): melatonin, melatonin+anaesthesia and melatonin+OHE and received melatonin (0.3 mg/kg, p.o.) on days –1, 0, 1, 2 and 3. Ten dogs were assigned to the control and OHE groups (*n* = 5) without melatonin treatment. OHE and anaesthesia were performed on day 0. Blood samples were obtained via jugular vein on days –1, 1, 3 and 5.

**Results:**

Melatonin and serotonin concentrations significantly increased in the melatonin, melatonin+OHE and melatonin+anaesthesia groups compared with the control group, while cortisol concentration decreased in the melatonin+OHE group compared with the OHE group. The concentrations of acute‐phase proteins (APPs) and inflammatory cytokines significantly increased after OHE. The CRP, SAA and IL‐10 concentrations decreased significantly in the melatonin+OHE group compared with the OHE group. The concentrations of cortisol, APPs and proinflammatory cytokines increased significantly in the melatonin+anaesthesia group compared with the melatonin group.

**Conclusions:**

The oral administration of melatonin before and after OHE help controlling the high levels of inflammatory APPs, cytokines and cortisol induced by OHE in female dogs.

## Graphical abstract

OHE surgery induced inflammation in female dogs. Oral melatonin administration before and after surgery can be useful to control the high levels of inflammatory acute‐phase proteins, cytokines and cortisol induced by OHE in female dogs.

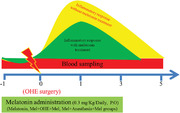

## 1 INTRODUCTION

Ovariohysterectomy (OHE) is a common surgery in veterinary medicine, but the stress induced by this surgery causes inflammation and changes in the concentrations of some hormones, such as cortisol. Melatonin, as an endogenous hormone, is secreted by the pineal gland rhythmically during the dark phase of the light‐dark cycle (Haldar, 2008). In addition to its circadian and antioxidant activities, melatonin has immune stimulatory effects, allowing it to act as a pro‐ or anti‐inflammatory regulator (Hardeland, 2019). Melatonin has been shown to have anti‐inflammatory effects in normal, pathologic and surgical conditions, such as cataract surgery (Sande et al., 2016). The action mechanism of melatonin is controlling IL‐6, IL‐4, IL‐1β, IFN‐ γ and TNF‐α as inflammatory cytokines and c‐reactive proteins (Farhadi et al., 2014; Kim et al., 2012).

Serotonin is a neurotransmitter with various regulatory functions in physiologic, emotional and behavioural processes. There is an association between aggressive behaviour and low levels of serum serotonin and 5‐hydroxyindoleacetic acid, the primary metabolite of serotonin in dogs, in the cerebrospinal fluids of dogs (Riggio et al., 2021). Serotonin is the key intermediate in melatonin synthesis from tryptophan (Zhao et al., 2019). Plasma serotonin concentrations were significantly lower in horses with surgical small intestinal colic compared with healthy control horses before, immediately after and the first morning after surgery (Torfs et al., 2015). Castration in dogs significantly decreased plasma serotonin concentration (Salavati et al., 2018). The assessment of serum serotonin levels during and after cardiac surgery showed no significant changes in plasma serotonin levels during or after cardiopulmonary bypass in 12 patients undergoing coronary artery graft surgery and five patients undergoing valve replacement (Anger & Prys‐Roberts, 1984).

As a well‐known stress marker in dogs, cortisol is produced by the adrenal gland, and its secretion is regulated through the hypothalamus‐hypophysis‐adrenal axis (Lensen et al., 2019). This hormone is involved in many physiologic and metabolic processes and plays a vital role in response to stress (Pineda & Dooley, 2008).

Trauma, infection, stress, neoplasia and inflammation activate inflammatory acute‐phase response as an early‐defence system. As part of this response, hepatocytes synthesise acute‐phase proteins, which have wide applications and high diagnostic value in cardiac and autoimmune diseases, organ transplantation and cancer treatment (Cray et al., 2009). After trauma, stress and inflammation, a local response induces proinflammatory cytokines, which will stimulate the liver and hepatocytes to produce acute‐phase proteins, leading to the initiation of the acute‐phase response, characterised by leucocytosis, complement activation, protease inhibition, clotting and opsonisation (Cray et al., 2009). CRP is an indicator of various pathologic conditions, such as infectious and autoimmune diseases, necrosis due to infarction and traumatic conditions; it can also increase or decrease cytokine production and movement of leucocytes from blood vessels (chemotaxis) (Cray et al., 2009; Pepys & Hirschfield, 2003). SAA can stimulate the migration of leucocytes, such as monocytes and T lymphocytes and alleviate inflammation (Cray et al., 2009). AGP binds to and inhibits endotoxins and can reduce neutrophils and the adverse effects on the complement system (Cray et al., 2009). While CRP and SAA are the major acute‐phase proteins in dogs, with a 10‐fold increase after an inflammatory stimulus, AGP is a moderate acute‐phase protein that increases 1‐ to 10‐fold following an inflammatory condition (Cray et al., 2009). There is an association between SAA and CRP levels and pyometra in dogs (Dąbrowski et al., 2007; Nakamura et al., 2008). CRP levels increased in various inflammatory conditions, such as acute pancreatitis, inflammatory bowel disease and suture removal (Jergens et al., 2003; Nakamura et al., 2008; Yamamoto et al., 1993).

This study investigated the effects of melatonin administration on the concentrations of acute‐phase proteins, inflammatory cytokines, serotonin and cortisol before and after OHE in female dogs. Furthermore, melatonin concentration was measured in all dogs during the study.

## 2 MATERIALS AND METHODS

### 2.1 Animals

Twenty‐five adult mixed‐breed and clinically healthy female dogs were selected in the anaestrous phase of the estrous cycle. The selected female dogs were aged 1–3 years and weighed 15–20 kg. Commercial dog food (Nutripet^TM^) was used to feed all dogs (300 g/dog/day). All dogs had free access to water and were kept in the same cage with a 12 h:12 h light/dark cycle. During the two weeks of preparation and adaptation, deworming and anti‐parasitic treatments were performed using tablets containing fenbendazole (150 mg), pyrantel embonate (144 mg) and praziquantel (50 mg). The overall health of dogs was evaluated daily during the study by checking their body temperature, heart rate, respiratory rate, appetite and behaviour while feeding and cleaning their shelter. All dogs were ovariohysterectomised at the end of the study and kept in a nongovernmental organisation shelter for adoption.

### 2.2 Experimental design

After the preparation period, the dogs were assigned into five groups (*n* = 5). The melatonin+OHE group was ovariohysterectomised at day 0 and received melatonin (0.3 mg/kg, p.o.; L'ORGANIQUE, Canada,) on days −1, 0, 1, 2 and 3 (Sande et al., 2016). The OHE group was ovariohysterectomised at day 0 but did not receive melatonin. The melatonin+anaesthesia group received anaesthesia without surgery on day 0 and melatonin on days −1, 0, 1, 2 and 3. The melatonin group received melatonin on days −1, 0, 1, 2 and 3. The control group neither underwent OHE nor received melatonin. Blood samples were collected before melatonin administration on days −1, 1, 3 and 5 from the jugular vein into plain glass tubes. The serum was separated by centrifugation for 10 min at 3000 rpm and stored at −20°C until laboratory analysis.

### 2.3 Surgical approach

Food and water deprivation was applied 12 h before the surgery. Acepromazine (0.05 mg/kg, i.m.) and xylazine (0.5 mg/kg, i.m.) were used as premedication; moreover, ketamine (5 mg/kg, i.v.) and diazepam (0.25 mg/kg, i.v.) were used as induction agents. Isoflurane (1.2%), vaporised in oxygen, was administered after intubation to maintain the general anaesthesia. Ampicillin (20 mg/kg, i.m.) and ketoprofen (1 mg/kg, i.m.) were injected at the end of surgery (Fossum et al., 2007). An anaesthesiologist carried out OHE with general anaesthesia and monitoring during the surgery.

### 2.4 Laboratory assay

A commercial canine solid‐phase sandwich ELISA kit (Shanghai Crystal Day Biotech Co., Ltd., China) was used to measure CRP (sensitivity 7.8 pg/mL, inter‐assay: CV<10%, intra‐assay: CV<8%), SAA (sensitivity 0.156 pg/mL, inter‐assay: CV<10%, intra‐assay: CV<8%;), tumour necrosis factor‐α (TNF‐α) (sensitivity 0.01 ng/L, inter‐assay: CV<10%, intra‐assay: CV<8%), interleukin‐1β (IL‐1β) (sensitivity 7.8 pg/mL, inter‐assay: CV<10%, intra‐assay: CV<8%), interleukin‐8 (IL‐8) (sensitivity 2.34 pg/mL, inter‐assay: CV<10%, intra‐assay: CV<8%), interleukin‐10 (IL‐10) (sensitivity 1.02 pg/mL, inter‐assay: CV<10%, intra‐assay: CV<8%) and serum melatonin (sensitivity 2.66 ng/mL, inter‐assay: CV<10%, intra‐assay: CV<8%). Alpha‐1‐acid glycoprotein (AGP) was measured by a commercial canine solid‐phase sandwich ELISA kit (Immunology Consultants Laboratory Inc., Portland, OR, USA; sensitivity 0.5 ng/mL, inter‐assay: CV<10%, intra‐assay: CV<8%). Serum serotonin was measured using a commercial solid‐phase sandwich ELISA kit (Labor Diagnostika Nord GmbH & Co., KG, Germany; sensitivity 10.2 ng/mL, inter‐assay‐CV<11%, intra‐assay‐CV<10%); also, serum cortisol was measured using another commercial solid‐phase sandwich ELISA kit (Monobind Inc., USA; sensitivity: 0.25 µg/dL, inter‐assay: CV<9.7%, intra‐assay: CV<8.2%). All laboratory methods and assays (kits) have been validated in the clinical pathology laboratory.

### 2.5 Statistical analysis

The Kolmogorov–Smirnov test evaluated the data distribution. For statistical significance and comparison, two‐way repeated‐measures ANOVA (with time, group and time/group interactions as the main factors), followed by post hoc Tukey's multiple comparisons), was performed using GraphPad Prism version 6 (GraphPad Software, San Diego, CA, USA). All data are presented as mean ± SEM. The level of significance was set at *p* < 0.05.

## 3 RESULTS

There was no significant difference between the study groups regarding the concentrations of acute‐phase proteins, inflammatory cytokines and hormones on day −1.

### 3.1 Melatonin

**FIGURE 1:**
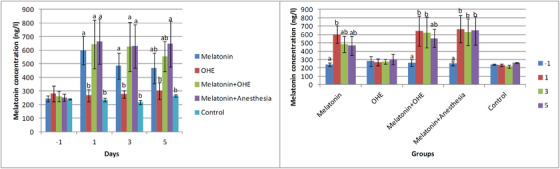
The mean ± SEM concentration of melatonin was compared between the study groups (i.e. melatonin, OHE, melatonin+OHE, melatonin+anaesthesia and control) on each sampling day (left) and between sampling days (i.e. −1, 1, 3, 5) in each group (right). Melatonin (0.3 mg/kg, p.o.) was administrated on days −1, 0, 1, 2 and 3. OHE and anaesthesia were performed on day 0. Different letters (a, b, c, d) above bars indicate significant differences (*p* < 0.05) between groups on each sampling day (left) and between sampling days in each group (right).

The time and group significantly affect melatonin concentration (*p* = 0.01), but their interaction did not significantly affect melatonin concentration. On days 1 and 3, melatonin concentration increased significantly in melatonin, melatonin+OHE and melatonin+anaesthesia compared to the control and OHE groups (*p* < 0.02). On day 5, melatonin concentration was significantly higher in the melatonin+anaesthesia group compared to the control and OHE groups (Figure 1). No significant difference was observed between the control and OHE groups on different sampling days (−1, 1, 3 and 5). There was a significant difference in melatonin concentration on day −1 versus day 1 (*p* = 0.02) in the melatonin group, on days 1 and 3 versus day −1 (*p* = 0.01) in the melatonin+OHE group and on days 1, 3 and 5 versus day −1 (*p* < 0.01) in the melatonin+anaesthesia group (Figure 1).

### 3.2 Cortisol

**FIGURE 2:**
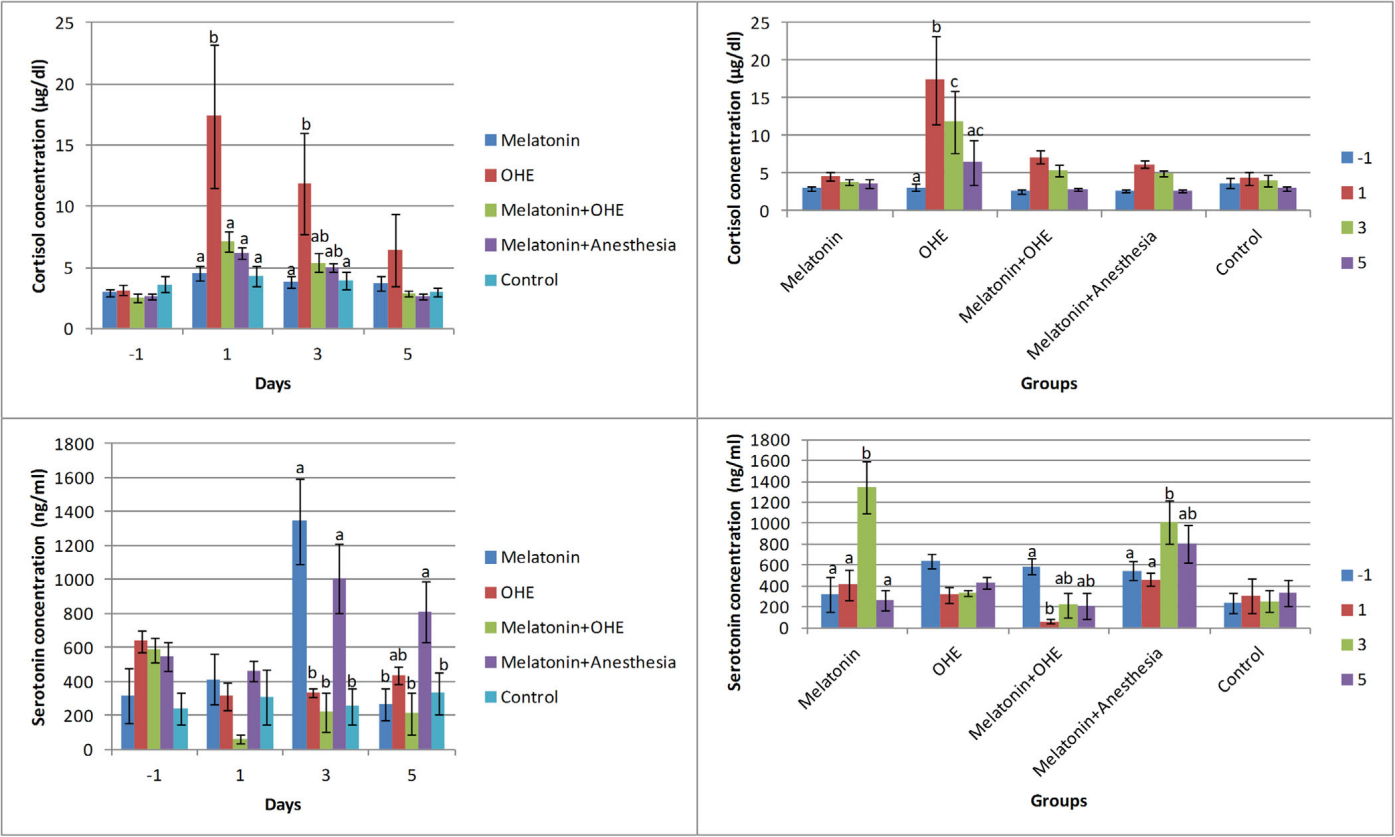
The mean ± SEM concentrations of cortisol and serotonin were compared between the study groups (i.e. melatonin, OHE, melatonin+OHE, melatonin+anaesthesia and control) on each sampling day (left) and between sampling days (i.e. −1, 1, 3, 5) in each group (right). Melatonin (0.3 mg/kg, p.o.) was administrated on days −1, 0, 1, 2 and 3. OHE and anaesthesia were performed on day 0. Different letters (a, b, c, d) above bars indicate significant differences (*p* < 0.05) between groups on each sampling day (left) and between sampling days in each group (right).

The time (*p* = 0.009) and group (*p* = 0.003) had significant effects on cortisol concentration, but the interaction between them did not significantly affect cortisol concentration. Cortisol concentration in the OHE group was significantly higher than in the other groups on day 1 but significantly lower in the melatonin group compared with the control group on day 3 (*p* = 0.02). Moreover, cortisol concentration in the OHE group was higher than in the control group on day 1 (*p* = 0.02). No significant difference was observed between the groups on day 5 (Figure 2). Cortisol concentration significantly increased on days 1 and 3 versus day −1 (*p* < 0.007) and decreased on day 3 versus day 1 (*p* < 0.005) in the OHE group (Figure 2).

### 3.3 Serotonin

Time, group and their interaction significantly affected serotonin concentration (*p* < 0.02). Serotonin concentration was significantly higher in the melatonin and melatonin+anaesthesia groups compared with the other groups on day 3 (*p* < 0.001). On day 5, serotonin concentration in the melatonin+anaesthesia group was significantly higher than that of the control, melatonin and melatonin+OHE groups (*p* < 0.04; Figure 2). No significant difference was observed between the control and OHE groups on different sampling days. Serotonin concentration increased on day 3 versus days −1, 1 and 5 in the melatonin group (*p* < 0.0001) and decreased on day 1 versus day −1 in the melatonin+OHE group (*p* = 0.01). Furthermore, serotonin concentration on day 3 was significantly higher than that on days 1 and −1 in the melatonin+anaesthesia group (*p* < 0.04; Figure 2).

### 3.4 C‐reactive protein (CRP)

**FIGURE 3:**
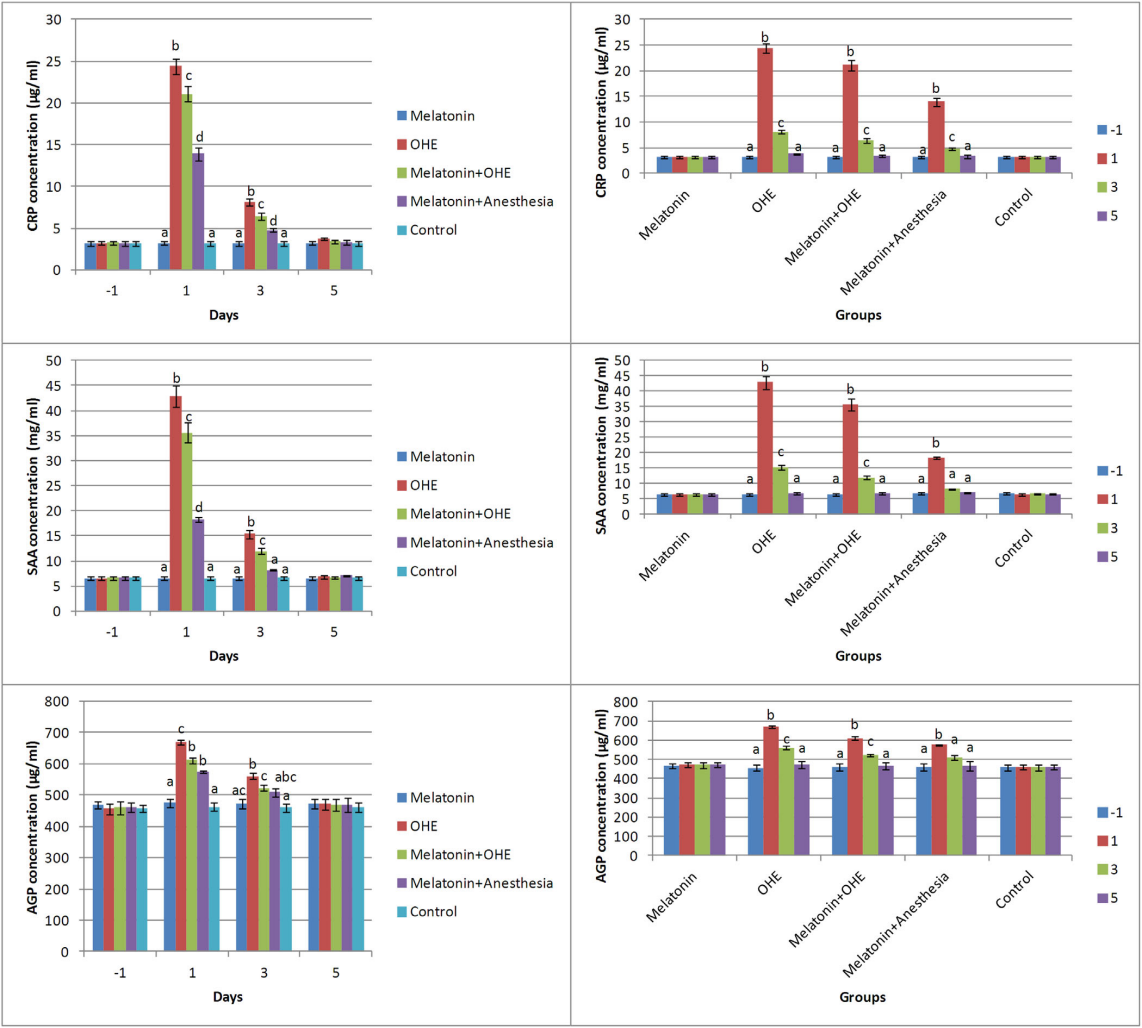
The mean ± SEM concentrations of CRP, SAA and AGP were compared between the study groups (i.e. melatonin, OHE, melatonin+OHE, melatonin+anaesthesia and control) on each sampling day (left) and between sampling days (i.e. −1, 1, 3, 5) in each group (right). Melatonin (0.3 mg/kg, p.o.) was administrated on days −1, 0, 1, 2 and 3. OHE and anaesthesia were performed on day 0. Different letters (a, b, c, d) above bars indicate significant differences (*p* < 0.05) between groups on each sampling day (left) and between sampling days in each group (right).

Time, group and their interaction significantly affected CRP concentration (*p* < 0.0001). Maximum CRP concentration was observed in OHE, melatonin+OHE and melatonin+anaesthesia groups, respectively, and the CRP concentration was significantly different between these groups on days 1 and 3 (*p* < 0.0001). CRP concentrations increased significantly in the OHE, melatonin+OHE and melatonin+anaesthesia groups compared with the melatonin and control groups (*p* < 0.0001; Figure 3). No significant changes were observed in CRP concentrations in the melatonin and control groups on different sampling days. CRP levels significantly increased on days 1 and 3 versus days −1 and 5 (*p* < 0.0001) and decreased on days 3 and 5 versus day 1 in the OHE, melatonin+OHE and melatonin+anaesthesia groups (*p* < 0.03; Figure 3).

### 3.5 Serum amyloid A (SAA)

Time, group and their interaction significantly affected SAA concentration (*p* < 0.0001). On day 1, a significant increase in SAA concentration was observed in the OHE, melatonin+OHE and melatonin+anaesthesia groups compared with control and melatonin groups (*p* < 0.001). On day 3, SAA concentration in the OHE group was significantly higher than in the melatonin+OHE group (*p* < 0.004). SAA concentration was significantly higher in the OHE and melatonin+OHE groups compared to the control, melatonin and melatonin+anaesthesia groups (*p* < 0.004; Figure 3). No significant changes in SAA concentrations were observed between different sampling days in the melatonin and control groups. SAA concentrations were significantly higher on day 1 versus day 3 in the OHE and melatonin+OHE groups, on days 1 and 3 versus days −1 and 5 in the OHE and melatonin+OHE groups (*p* < 0.0001), and on day 1 versus other sampling days in the melatonin+anaesthesia group (Figure 3).

### 3.6 Alpha‐1‐acid glycoprotein (AGP)

Time, group and their interaction significantly affected AGP concentration (*p* < 0.0001). AGP concentration was higher in the OHE group compared with other groups on day 1. AGP concentrations increased significantly in the melatonin+OHE and melatonin+anaesthesia groups compared with the control and melatonin groups on day 1 (*p* < 0.001). On day 3, AGP concentration was significantly higher in the OHE group compared with the control, melatonin and melatonin+OHE groups (*p* < 0.002) and in the melatonin+OHE group compared with the control group (*p* = 0.01; Figure 3). AGP concentrations in the OHE and melatonin+OHE groups significantly increased on days 1 and 3 versus days 1 and 5 and decreased on days 3 and 5 versus day 1 (*p* < 0.02). Moreover, AGP concentration in the melatonin+anaesthesia group increased significantly on day 1 versus days −1, 3 and 5 (*p* < 0.004; Figure 3).

### 3.7 Tumour necrosis factor‐α (TNF‐α)

**FIGURE 4:**
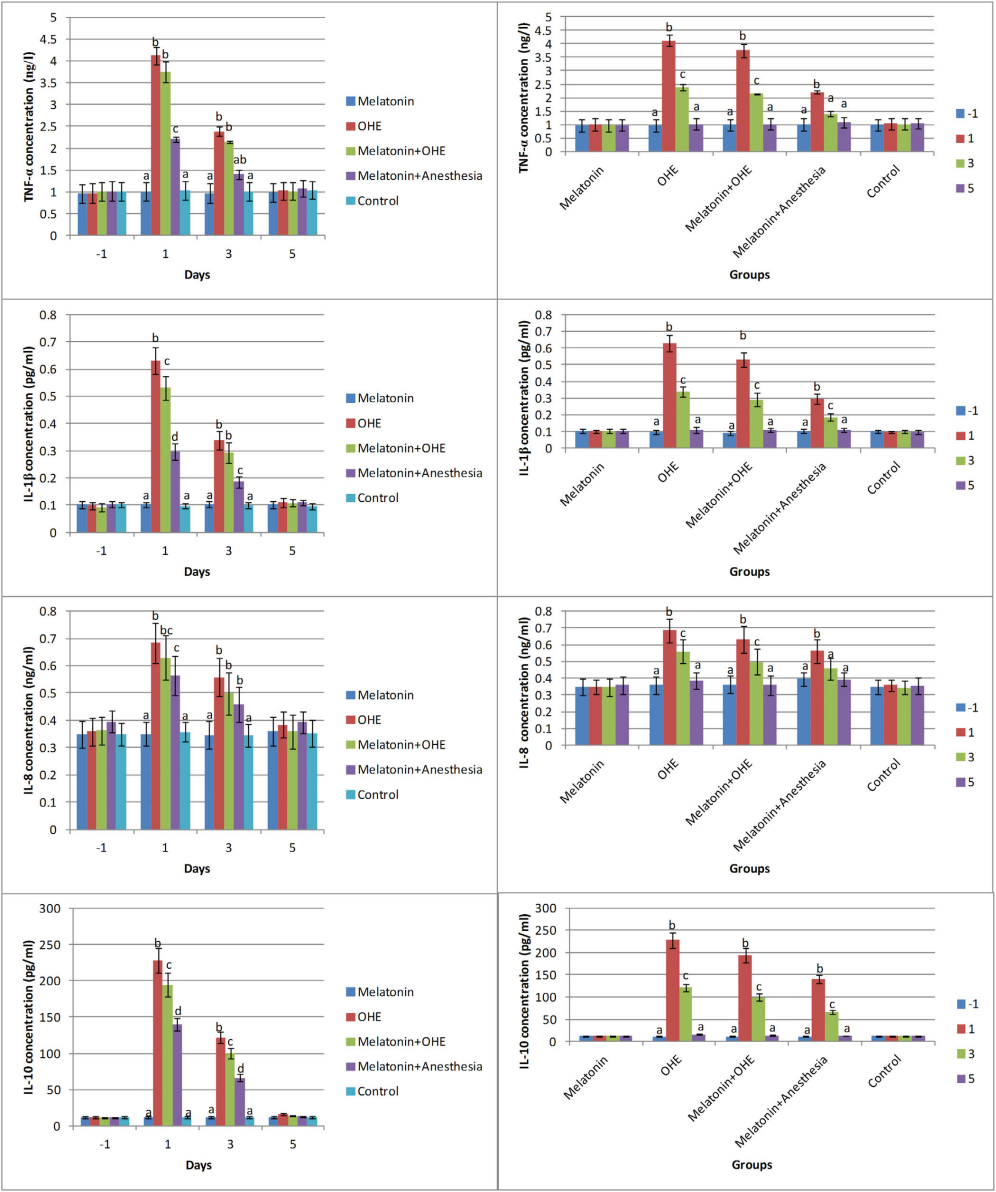
The mean ± SEM concentrations of TNF‐α, IL‐1β, IL‐8 and IL‐10 were compared between the study groups (i.e. melatonin, OHE, melatonin+OHE, melatonin+anaesthesia and control) on each sampling day (left) and between sampling days (i.e. −1, 1, 3, 5) in each group (right). Melatonin (0.3 mg/kg, p.o.) was administrated on days −1, 0, 1, 2 and 3. OHE and anaesthesia were performed on day 0. Different letters (a, b, c, d) above bars indicate significant differences (*p* < 0.05) between groups on each sampling day (left) and between sampling days in each group (right).

Time, group and their interaction between them significantly affected TNF‐α concentration (*p* < 0.0001). TNF‐α concentration was significantly higher in the OHE and melatonin+OHE groups compared with the other groups on day 1 (*p* < 0.0001). In addition, on day 1, TNF‐α concentrations significantly increased in the melatonin+anaesthesia group compared with the control and melatonin groups. They decreased in the melatonin+anaesthesia group compared with the OHE and melatonin+OHE groups (*p* < 0.0001). On day 3, TNF‐α concentration was significantly higher in the OHE group compared with the other groups and in the melatonin+OHE group compared with the control and melatonin groups (*p* < 0.005; Figure 4). TNF‐α concentrations in the melatonin and control groups were not significantly different on different sampling days. TNF‐α concentrations increased significantly on day 1 versus day 3 and days 1 and 3 versus days −1 and 5 in the OHE and melatonin+OHE groups (*p* < 0.0007). In addition, TNF‐α concentration increased significantly on day 1 versus other sampling days in the melatonin+anaesthesia group (*p* < 0.02; Figure 4).

### 3.8 Interleukin‐1β (IL‐1β)

Time, group and their interaction between them significantly affected IL‐1β concentration (*p* < 0.0001). IL‐1β concentration was significantly higher in the OHE, melatonin+OHE and melatonin+anaesthesia groups compared to the control and melatonin groups on day 1. The maximum concentration of IL‐1β was observed in the OHE group compared to the melatonin+OHE and melatonin+anaesthesia groups on day 1 (*p* < 0.0001). IL‐1β concentration was significantly higher in the OHE, melatonin+OHE and melatonin+anaesthesia groups compared to the control and melatonin groups on day 3 (*p* < 0.0003; Figure 4). There was no significant difference in IL‐1β concentration between the melatonin and control groups on different sampling days. IL‐1β concentration significantly increased on days 1 and 3 versus days −1 and 5 and decreased on day 3 versus day 1 in the OHE, melatonin+OHE and melatonin+anaesthesia groups (*p* < 0.0005; Figure 4).

### 3.9 Interleukin‐8 (IL‐8)

Time (*p* = 0.02) and the interaction between time and group (*p* < 0.0001) had a significant effect on IL‐8 concentration. IL‐8 concentration was significantly higher in the OHE, melatonin+OHE and melatonin+anaesthesia groups compared with the control and melatonin groups on day 1. The highest concentration of IL‐8 was observed in the OHE group compared with the melatonin+anaesthesia group (*p* < 0.0001). IL‐8 concentration was significantly higher in the OHE, melatonin+OHE and melatonin+anaesthesia groups compared with the control and melatonin groups on day 3 (*p* < 0.003; Figure 4). No significant difference was observed in IL‐8 concentration between the melatonin and control groups on different sampling days. IL‐8 concentration significantly increased on days 1 and 3 versus days −1 and 5 and decreased on day 3 versus day 1 in the OHE and melatonin+OHE groups (*p* < 0.008). IL‐8 concentration significantly increased on day 1 versus other sampling days in the melatonin+anaesthesia group (*p* < 0.03; Figure 4).

### 3.10 Interleukin‐10

Time, group and the interaction between them significantly affected IL‐10 concentration. An IL‐10 concentration was significantly higher in the OHE, melatonin+OHE and melatonin+anaesthesia groups compared with the control and melatonin groups. Also, a significant increase in IL‐10 concentration was observed in the OHE, melatonin+OHE and melatonin+anaesthesia groups, respectively, on days 1 and 3 (*p* < 0.0001; Figure 4). No significant difference in IL‐10 concentration was observed in the melatonin and control groups on different sampling days. IL‐10 concentrations significantly increased on days 1 and 3 versus days −1 and 5 and decreased on day 3 versus day 1 in the OHE, melatonin+OHE and melatonin+anaesthesia groups (*p* < 0.0001; Figure 4).

## 4 DISCUSSION

### 4.1 Hormones (melatonin, cortisol and serotonin)

In the present study, the serum melatonin concentration increased following daily oral melatonin administration (0.3 mg/kg) in intact and ovariohysterectomised female dogs. There was a correlation and dose dependency between the oral administration of melatonin and its bioavailability in dogs. Furthermore, it was reported that first‐pass hepatic metabolism did not affect the bioavailability of oral melatonin and that an oral dose of 10 mg/kg of melatonin showed a high absolute bioavailability in dogs (Yeleswaram et al., 1997). Increasing doses of oral melatonin linearly increased serum levels, indicating that melatonin was absorbed from the gastrointestinal tract by a nonsaturated transport system (probably passive transport) (Sääf et al., 1980).

Surgical stress increased cortisol concentration in the OHE group. However, melatonin administration decreased cortisol concentration in the melatonin+OHE group compared with the OHE group. Oral melatonin administration did not affect cortisol concentration in intact female dogs. Two possible explanations have been suggested to be responsible for the effect of melatonin on reducing cortisol concentration: (a) melatonin increases serotonin concentration, and increased serotonin, in turn, decreases cortisol concentration in animals with aggressive behaviour and during stress conditions (Riggio et al., 2021) and (b) it is possible that melatonin reduces cortisol secretion and concentration through its receptors in the adrenal gland (Frank et al., 2004).

In this study, cortisol concentration increased after OHE. In another study, the effects of analgesia and anaesthesia on serum cortisol concentrations were determined in six female dogs undergoing OHE. The groups included in this study were control, analgesia with butorphanol, anaesthesia with thiopentone sodium, halothane and oxygen and anaesthesia plus surgery. In addition, the adrenocorticotropic hormone (ACTH) challenge was performed for each of the four groups. A temporary increase in serum cortisol concentrations was observed in all groups, but cortisol concentrations in the group undergoing OHE were four times higher than presurgery levels in other groups. This increase in cortisol concentrations lasted about 6 h and then returned to pretreatment levels after 24 h (Fox et al., 1994). The marked increase in cortisol concentrations in the surgery group was probably due to nociceptor (a pain receptor) inputs associated with OHE. Injury at the site of surgical incision produced neural impulses, which acted via the hypothalamus and mediated stress response to surgery in humans and laboratory animals (Anand et al., 1987; Morishima et al., 1980; Sanhouri et al., 1991).

In the present study, anaesthesia, probably due to the stress it induced, increased cortisol concentration in the melatonin+anaesthesia group compared to the melatonin group. Similarly, in a study on female dogs (Fox et al., 1994), the excitement induced during the second stage of anaesthesia was attributed to the increase in cortisol concentration in the anaesthesia group; this increase in cortisol concentration following anaesthesia was similar to what occurred to humans and decreased after a while (Taylor, 1988). In the same study, it was stated that although anaesthesia with halothane in horses led to cortisol release due to hypotension and hypoxia, as two potent stimulants (Taylor, 1989), it did not stimulate cortisol release in female dogs.

In this study, female dogs received melatonin on days −1, 0, 1, 2 and 3, and sampling was performed on days −1, 1, 3 and 5. The results showed that serotonin concentration increased until day 3 but decreased on day 5 (48 h after the last melatonin administration) versus day 3 in female dogs that received melatonin. Similarly, in another study, the intraperitoneal administration of melatonin to rats increased serotonin concentration in the midbrain within 20 min. The mechanisms involved were not clear, but it was suggested that melatonin might alter the function of neurons containing serotonin or increase serotonin concentration in the brain by inhibiting the release of the amine, increasing serotonin reuptake or preventing serotonin intra‐ or extraneural metabolism (Anton‐Tay et al., 1968). In another study on rats, an increase in serotonin concentration was observed in the medial hypothalamus, a part of the brain with serotoninergic terminals, 1 and 1.5 h after melatonin administration at a dose of 0.5 mg/kg. When the melatonin dosage was increased to 1 mg/kg, serotonin levels increased in other brain parts, including the preoptic area‐anterior hypothalamus, medial and posterior hypothalamus, amygdala and midbrain. These findings suggested that specific brain regions may be sensitive to melatonin administration in a dose‐dependent manner (Miguez et al., 1994).

In the present study, melatonin administration in nonsterilised female dogs increased serotonin concentration compared to the control, OHE and melatonin+OHE groups on day 3. OHE did not significantly reduce serotonin concentration compared to the control group. Melatonin administration before and after OHE did not increase serotonin concentration significantly compared to the OHE group. A significant difference was observed between the melatonin+anaesthesia group, on the one hand, and the control and melatonin+OHE groups, on the other hand, regarding serotonin concentration on days 3 and 5. However, there are conflicting results regarding the effect of anaesthesia on melatonin and serotonin concentrations. Some studies, for instance, have reported increased plasma melatonin concentration after isoflurane and propofol anaesthesia, while others have reported a decrease or no significant changes in melatonin concentration after anaesthesia (Kärkelä et al., 2002; Nishimura et al., 1998; Reber et al., 1998). These discrepant results may be due to the differences in surgical procedures, premedication, anaesthetic techniques and methods used to measure melatonin concentration (Naguib et al., 2007).

### 4.2 Acute‐phase protein and inflammatory cytokines

The systemic immune response to a traumatic procedure begins with the stimulation of macrophages and granulocytes, leading to the release of different inflammatory cytokines, such as IL‐6, IL‐1β and TNF‐α. Pyometra and OHE increase CRP (a major acute‐phase protein in dogs) and SAA concentrations. These two positive acute‐phase proteins are reliable markers for assessing the complications in the postoperative period of OHE (Cerón et al., 2005; Dąbrowski et al., 2007).

The molecular mechanism responsible for the anti‐inflammatory properties of melatonin is applied through the regulation of different molecular pathways, which, in turn, inhibits the generation of excessive amounts of nitric oxide. Nitric oxide plays a vital role in inducing inflammation and increasing the levels of leucotrienes and prostanoids. It was also shown that regulation of molecular pathways inhibited the expression of isoforms of inducible nitric oxide synthase and cyclooxygenase and prevented the overproduction of mediators of the inflammatory process, such as cytokines, chemokines and adhesion molecules (Mauriz et al., 2013).

Phacoemulsification is a surgical treatment for cataract, but it is associated with degrees of postoperative inflammation. Compared to dexamethasone and nonsteroidal anti‐inflammatory drugs, melatonin administration has been shown to be effective in controlling the inflammatory response after phacoemulsification surgery in dogs (Sande et al., 2016). Melatonin administration in healthy dogs did not significantly alter the expression of IL‐2 or interferon‐gamma (Peace et al., 2019). Similarly, the results of the present study revealed that melatonin administration did not change the concentrations of IL‐8, IL‐10 and IL‐1β in healthy dogs. Furthermore, it has been reported that melatonin treatment effectively decreases acute‐phase proteins and cytokines, including SAA, CRP, IL‐1β and TNF‐α, in castrated dogs but not in intact dogs (Nazifi et al., 2020).

In this study, CRP, SAA and AGP concentrations increased after OHE due to the inflammation induced by surgery, and their concentrations decreased to the control group level by day 5. CRP and SAA concentrations were significantly lower in the melatonin+OHE group compared with the OHE group on days 1 and 3 (the last day of melatonin treatment). CRP concentration on days 1 and 3 and SAA and AGP concentrations on day 1 were higher in the melatonin+anaesthesia group compared with the melatonin group. Intravenous general anaesthesia with propofol and remifentanil in human patients increased IL‐6, IL‐7, IL‐8 (a major factor in acute inflammation), IL‐15 and other biomarkers such as monocyte chemoattractant protein 1, placental growth factor, macrophage inflammatory protein‐1β and vascular endothelial growth factor‐A. The neuroinflammatory response was dominated by IL‐6, IL‐8 and modulatory calcineurin interacting with protein‐1 (Pikwer et al., 2017; Tang et al., 2011). In another study, inflammatory biomarkers in the cerebrospinal fluid were evaluated after anaesthesia and the results showed a strong neuroinflammatory response with an increase in IL‐6, TNF‐α and IL‐10 concentrations (Tang et al., 2011). The higher incidence of postoperative cognitive dysfunction in patients undergoing surgery under sevoflurane anaesthesia compared with those undergoing surgery under intravenous propofol anaesthesia was associated with significantly higher plasma levels of IL‐6 and TNF‐α within the first week after anaesthesia (Qiao et al., 2015). An approximately 60‐min exposure to isoflurane general anaesthesia, without induced surgical stress, caused a significant increase in serum IL‐1β concentration, as a marker of systemic inflammation, in children undergoing MRI (Whitaker et al., 2017). In the present study, a significant increase was observed in TNF‐α, IL‐1β, IL‐10 and IL‐8 concentrations in the OHE compared with the control group due to the inflammatory effects of surgery. However, a significant decrease was observed in IL‐1β concentration on day 1 and IL‐10 concentration on days 1 and 3 (the last day of melatonin administration) in the melatonin+OHE group compared with the OHE group. TNF‐α, IL‐1β and IL‐10 concentrations significantly increased in the melatonin+anaesthesia group compared with the melatonin group, probably due to the effects of anaesthetic drugs.

Strengthening body defence and avoiding the depression of immune responses that leads to inflammation helps treat different insulting conditions such as surgical trauma. Melatonin's anti‐inflammatory, antioxidant and immunomodulatory effects can balance the stress conditions to the body`s benefit. One of the mechanisms of melatonin is to preserve mitochondria's structural and functional integrity. Melatonin is synthesised in high concentration and functions in mitochondria. Melatonin deficiency leads to the release of cytochrome C and damage‐associated molecular pattern molecules, such as mtDNA and cardiolipin, which initiate apoptosis or inflammation. Furthermore, low melatonin allows the cell to switch from respiratory metabolism to anaerobic glycolysis. Glycolysis cannot generate acetyl‐coenzyme A, a cofactor of melatonin synthesis. This repeated defective cycle increases cell and body damage (Tan & Hardeland, 2020). So, supplementation of melatonin before and after surgery can inhibit this defective cycle, reduce oxidative stress and inflammation and accelerate recovery.

## 5 CONCLUSIONS

Melatonin administration in intact and ovariohysterectomised female dogs increased serum melatonin concentration. OHE increased the concentrations of cortisol, acute‐phase proteins (i.e. SAA, CRP and AGP) and inflammatory cytokines (i.e. TNF‐α, IL‐8, IL‐10 and IL‐1β). The oral administration of melatonin before and after OHE significantly decreased cortisol, CRP, SAA and IL‐10 concentrations but increased serotonin concentration. However, oral melatonin did not affect the concentrations of inflammatory proteins and cytokines in intact female dogs.

## AUTHOR CONTRIBUTIONS

Sina Salavati: conceptualisation; data curation; investigation; methodology; resources; writing – original draft. Asghar Mogheiseh: conceptualisation; data curation; funding acquisition; investigation; methodology; project administration; resources; supervision; writing – original draft; writing – review & editing. Saeed Nazifi: conceptualisation; funding acquisition; investigation; methodology; resources; supervision; validation; writing – original draft; writing – review & editing. Atefeh Amiri: data curation; methodology; visualisation.

## CONFLICT OF INTEREST STATEMENT

The authors have no conflict of interest to declare.

## ETHICS STATEMENT

Experimental protocols were performed in accordance with the Iranian animal ethics framework under the supervision of the Iranian Society for the Prevention of Cruelty to Animals and Shiraz University Research Council (IACUC no: 4687/63). The recommendations of European Council Directive (2010/63/EU) of September 22, 2010, regarding the standards in the protection of animals used for experimental purposes, were also followed.

### PEER REVIEW

The peer review history for this article is available at https://publons.com/publon/10.1002/vms3.1112.

## Data Availability

The data that support the findings of this study are available from the corresponding author upon reasonable request.
